# Comparison of Neoadjuvant Chemotherapy, Chemoimmunotherapy, and Upfront Radical Cystectomy in High-Risk Bladder Cancer: A Single-Center Retrospective Study

**DOI:** 10.32604/or.2026.083704

**Published:** 2026-09-14

**Authors:** Shaohua Chen, Xiao Gan, Ping Lv, Qinggui Meng, Xiaocao Lin, Qingyun Zhang

**Affiliations:** 1 Department of Urology, Guangxi Medical University Cancer Hospital, Nanning, China; 2 Department of Urology, Wuzhou Workers’ Hospital, Wuzhou, China

**Keywords:** Muscle-invasive bladder cancer (MIBC), radical cystectomy, neoadjuvant chemotherapy (NAC), immune checkpoint inhibitors (ICIs), ICIs with chemotherapy (NAC-ICI), pathological complete response (pCR), treatment-related adverse events (TRAEs)

## Abstract

**Background:** While radical cystectomy is standard for muscle-invasive bladder cancer (MIBC), micrometastasis-related recurrence is common. Cisplatin-based neoadjuvant therapy (NAT) confers modest benefits, while immune checkpoint inhibitors (ICIs) provide new efficacy-enhancing strategies. This study aims to compare the efficacy and safety of ICIs with chemotherapy (NAC-ICI), neoadjuvant chemotherapy (NAC), and no neoadjuvant therapy (NNAT). It also explores potential biomarkers predictive of NAT response and evaluates the real-world efficacy of NAC-IC. **Method:** This single-center retrospective analysis included 80 radical cystectomy patients, with 51 NNAT group and 29 in the NAT group. Survival outcomes were evaluated by Kaplan-Meier survival curves, with log-rank test and restricted mean survival time (RMST) for comparisons. Pathological responses were assessed by complete response (pCR) and downstaging (pDS). Treatment-related adverse events (TRAEs) and predictive hematological biomarkers were analyzed. **Results:** Kaplan-Meier curves showed NAT is non-significant survival advantage over NNAT (*p* = 0.30). The RMST analysis showed the NAC-ICI had significantly longer RMST than the NNAT (56.00 vs. 47.73 months; Δ = +8.27 months; *p* = 0.0008). Fisher’s exact test showed that NAC-ICI had a numerically higher pCR rate (42.10%) than NAC (20.00%), but the difference was non-significant (*p* = 0.414) ROC analysis showed baseline platelet count (PLT) was a significant negative predictor of pCR after NAC-ICI. **Conclusions:** This study indicates potential feasibility and safety of NAT for MIBC patients. Chemoimmunotherapy trends toward better pathological and survival outcomes than chemotherapy alone, but no statistically significant differences were observed. Baseline PLT may be a potential prdictive biomarker.

## 1 Introduction

As is well documented, urothelial carcinoma (UC) arises from the urothelial lining of the urinary tract and includes upper tract UC (UTUC), originating from the renal pelvis or ureter, and lower urinary tract UC [1], predominantly bladder cancer, which constitutes the vast majority of UC cases. Indeed, bladder cancer accounts for over 90% of all UC diagnoses and is among the most prevalent malignancies of the urinary system [2]. Globally, it ranks ninth in cancer incidence, with males affected approximately three times more often than women [3]. According to the depth of tumor invasion, bladder cancer is classified into non-muscle-invasive bladder cancer (NMIBC) and muscle-invasive bladder cancer (MIBC) [4]. At the time of diagnosis, 10–15% of MIBC patients already harbor clinically undetectable micrometastases [5]. Although radical cystectomy with pelvic lymph node dissection (PLND) remains the standard treatment for MIBC, it exclusively targets macroscopic disease and cannot eradicate micrometastases that may have already disseminated through hematogenous or lymphatic pathways [6,7]. These residual tumor cells contribute to distant relapse in up to 50% of patients, resulting in a 5-year overall survival (OS) rate of below 50% [8]. Such outcomes highlight the limitations of surgery alone and the need for effective systemic approaches to improve long-term survival in MIBC.

Recently, neoadjuvant therapy (NAT), administered prior to definitive surgery, has emerged as a promising strategy to address the limitations of surgery alone in MIBC [9]. Among NAT modalities, cisplatin-based neoadjuvant chemotherapy (NAC), most commonly the gemcitabine-cisplatin (GC) regimen, remains the standard of care, achieving a pathological complete response (pCR) rate of up to 38% [10]. However, its capacity to eradicate micrometastatic disease remains suboptimal, as evidenced by recurrence or death in over 40% of cases within three years [11]. The advent of immune checkpoint inhibitors (ICIs), particularly agents targeting the programmed cell death protein 1/programmed death-ligand 1 (PD-1/PD-L1) axis, has transformed the therapeutic landscape across multiple solid tumors [12,13]. In the neoadjuvant setting, combining ICIs with chemotherapy (NAC-ICI) provides a synergistic approach by enhancing anti-tumor immunity and delivering direct cytotoxic effects [14]. This strategy can improve pCR rates, enhance micrometastatic clearance, minimize the risk of postoperative distant metastasis, and prolong disease-free survival, particularly in high-risk populations [15,16].

For instance, tislelizumab has emerged as one of the most promising ICIs in this setting. This humanized IgG4 monoclonal antibody, engineered with a modified Fc region, limits antibody-dependent cellular phagocytosis while enhancing direct tumor killing activities [17]. It also promotes memory T cell generation [18] and demonstrates higher PD-L1 binding affinity than pembrolizumab or nivolumab [2]. With outstanding efficacy and safety across multiple solid tumors, including UC [19,20], tislelizumab represents a candidate for neoadjuvant use in MIBC. Toripalimab, developed as China’s first PD-1 antibody, has demonstrated promising efficacy and safety in UC as well as other malignancies, providing a cost-effective alternative to imported agents [21,22,23]. Although evidence remains preliminary, it provides an alternative option in the Chinese clinical context. Collectively, both tislelizumab and toripalimab offer promising neoadjuvant strategies for cisplatin-ineligible patients and may support bladder-preserving approaches, shifting treatment goals from radical resection toward a balance between oncologic control and organ preservation [24,25].

Nevertheless, challenges persist in the clinical application of tislelizumab- or toripalimab-based neoadjuvant chemotherapy immunotherapy, including variability in patient responses, insufficient long-term safety data, and the absence of standardized protocols for integrating ICIs with chemotherapy [26,27,28]. These uncertainties emphasize the need for real-world evidence to validate clinical trial results, refine patient selection, and guide regimen optimization. To address these gaps, this study was a single-center retrospective cohort study involving 80 patients with bladder cancer (clinical stage cT1–T4a, with high-risk T1 cases selected according to predefined criteria), all of whom underwent radical cystectomy. 

We aimed to compare the efficacy and safety of three treatment strategies: NAC-ICI, NAC, and upfront radical cystectomy without NAT. The study also aimed to explore potential biomarkers predictive of NAT response, and assess the real-world clinical performance of NAC-ICI in routine practice.

## 2 Materials and Methods

### 2.1 Study Design and Endpoints

This was a single-center retrospective cohort study conducted to evaluate the efficacy and safety of NAT in MIBC patients. The study endpoints were defined as follows: pCR was defined as ypT0N0M0, and pathological downstaging (pDS) as any reduction in pathologic stage following NAT compared with the initial clinical stage (ypTNM < cTNM). OS was defined as the time from radical cystectomy to death from any cause. Postoperative complications were classified according to the European Perioperative Clinical Outcome (EPCO) definitions [29] and treatment-related adverse events (TRAEs) were assessed using the National Cancer Institute Common Terminology Criteria for Adverse Events (CTCAE), version 5.0. The primary endpoints were pCR and pDS in patients receiving NAT. Secondary endpoints included OS in all patients, postoperative complications in all patients, and TRAEs in patients receiving NAT. The study was approved by the Guangxi Medical University Cancer Hospital Ethical Review Committee (No. KY20251027) and adhered to the Declaration of Helsinki. All patients provided written informed consent.

### 2.2 Inclusion and Exclusion Criteria

The inclusion criteria were as follows: (i) histologically confirmed urothelial carcinoma diagnosed from transurethral resection of bladder tumor (TURBT) specimens; (ii) clinical stage cT1–T4a, N0–Nx, M0, according to the eighth edition of the American Joint Committee on Cancer (AJCC) staging system, with no evidence of distant metastasis on preoperative imaging. Consistent with the European Association of Urology (EAU) guidelines on non-muscle-invasive bladder cancer, cT1 disease was included only if meeting high-risk or very high-risk criteria, defined as the presence of high-grade histology, carcinoma *in situ* (CIS), variant histologies (micropapillary, plasmacytoid, sarcomatoid, squamous, or glandular differentiation), multifocality, or uncontrollable hematuria requiring radical cystectomy after TURBT; cT2–T4a disease was included regardless of additional risk factors; (iii) eligibility for cisplatin-based chemotherapy and adequate performance status for radical cystectomy, including patients eligible for NAC-ICI; (iv) absence of prior systemic chemotherapy or immunotherapy; and (v) adequate hepatic and renal function. 

The exclusion criteria were as follows: (i) contraindication due to cisplatin allergy or severe cisplatin-related adverse reactions; (ii) presence of severe hepatic/renal insufficiency, cardiopulmonary impairment, or immune system disorders; (iii) presence of other malignancies or prior antineoplastic therapy; (iv) pregnancy or lactation; and (v) non-adherence to follow-up regimens.

### 2.3 Treatment Regimens

Treatment allocation was not randomized. Patients received neoadjuvant therapy (NAC or NAC-ICI) or underwent upfront radical cystectomy (NNAT) based on clinical judgment, performance status, tumor characteristics, and patient preference after shared decision-making. In the no neoadjuvant therapy (NNAT) group, patients underwent radical cystectomy alone. In the NAC group, patients received four cycles of cisplatin- or carboplatin-based chemotherapy, consisting of gemcitabine (1000 mg/m^2^ on days 1 and 8 of each cycle; Qilu Pharmaceutical Co., Ltd., Haikou, Hainan, China) combined with either cisplatin (70 mg/m^2^ on day 2; Jiangsu Hansoh Pharmaceutical Group Co., Ltd., approval No. H20040813, Lianyungang, Jiangsu, China) or carboplatin (area under the concentration-time curve = 4 on day 2; Qilu Pharmaceutical Co., Ltd., approval No. H20020180, Jinan, Shandong, China). Each cycle lasted 21 days. In the NAC-ICI group, patients received four cycles of NAC-ICI, composed of gemcitabine (1000 mg/m^2^ on days 1 and 8), cisplatin (70 mg/m^2^ on day 2) or carboplatin (area under the concentration-time curve = 4 on day 2), and either tislelizumab (200 mg on day 4; BeiGene Co., Ltd., approval No. S20190045, Guangzhou, Guangdong, China) or toripalimab (240 mg on day 4; Shanghai Junshi Biosciences Co., Ltd., approval No. S20180015, Shanghai, China). Each cycle also lasted 21 days. Tumor status was assessed after each treatment cycle by computed tomography (CT) and clinical evaluation, and radical cystectomy was performed immediately rather than after completion of all four planned cycles in patients with disease progression.

### 2.4 Data Collection and Definitions

Hematological parameters were measured using a standard automated hematology analyzer following routine clinical laboratory protocols; the specific instrument details were not available from the retrospective records. All hematological parameters were assessed within one day prior to the initiation of NAT. Several composite indices with potential predictive value for NAT response were then calculated, including: neutrophil-to-lymphocyte ratio (NLR), platelet-to-lymphocyte ratio (PLR), systemic immune-inflammation index (SII, defined as [platelet count × neutrophil count]/lymphocyte count), monocyte-to-lymphocyte ratio (MLR), lymphocyte-to-monocyte ratio (LMR), and corrected platelet-to-lymphocyte ratio (cPLR), calculated as the relative change in PLR from baseline, i.e., (PLR at post-therapy − PLR at pre-therapy)/pre-therapy PLR, where post-therapy was defined as within 7 days after completion of all planned cycles of NAT. Pathological data were collected from TURBT biopsy specimens before NAT and from postoperative resection specimens, including tumor stage and histologic subtype. Pathological assessments were performed independently by two experienced uropathologists who were blinded to the treatment groups; any disagreements were resolved by consensus.

### 2.5 Statistical Analysis

Statistical analyses were performed using R version 4.4.1. Normally distributed continuous variables were compared using *t*-tests or one-way ANOVA, whereas non-normally distributed continuous variables were compared using the Mann-Whitney U test or Kruskal-Wallis H test. Categorical variables were compared using the chi-square test or Fisher’s exact test. The Kaplan-Meier method was used to estimate survival and plot survival curves, and differences among subgroups were compared using the Log-rank test. Because median survival was not reached and the number of survival events was limited, the proportional hazards assumption for Cox regression was not reliably met. Therefore, restricted mean survival time (RMST) up to the common follow-up horizon was estimated to compare survival between groups. The predictive performance of baseline platelet count for achieving pCR was evaluated using the receiver operating characteristic (ROC) curve, with the optimal cut-off value determined by maximizing Youden’s index (J = Sensitivity + Specificity − 1). Two-sided *p* values < 0.05 were considered statistically significant.

## 3 Results

### 3.1 Baseline Clinicopathological Characteristics of the Study Cohort

To assess the comparability of the included patients, baseline clinical and pathological features between the NNAT and NAT cohorts were analyzed, as shown in Table 1. A total of 80 eligible patients were included, including 51 in the NNAT group and 29 in the NAT group. The median age was 67.5 years, with an interquartile range (IQR) of 58.0–72.5 years, and most patients were male (86.3%). The mean body mass index (BMI) was 22.3 ± 3.3 kg/m^2^, and there was no significant difference between the two groups in age, BMI, sex distribution, or smoking status. Regarding comorbidities, 36.3% of patients had hypertension and 13.8% had diabetes, while 25.0% presented with hydronephrosis at baseline. Histopathological analysis revealed that pure urothelial carcinoma accounted for 81.3% of the total cohort, with similar distributions between the NNAT (74.5%) and NAT (93.1%) groups. Urothelial carcinoma with variant differentiation (squamous, glandular, or plasmacytoid) was observed in 13.7% of patients, while pure variant or mixed carcinomas (including carcinoma *in situ*, pure squamous cell carcinoma, small cell carcinoma, and sarcomatoid carcinoma) accounted for 5.0%. Among the 65 patients with pure urothelial carcinoma, 17 had T1 disease and 48 had MIBC (T2–T4); 5 of these had concurrent CIS as a non invasive component. Detailed distributions of variant differentiation subtypes are presented in Table 1 [30]. The distribution of urinary diversion types was similar between the two cohorts. Within the NAT group, 93.1% received the GC regimen, and the remaining 6.9% received the gemcitabine-carboplatin (GCa) regimen. For immunotherapy, tislelizumab or toripalimab was used in 65.5% of NAT patients. Overall, baseline demographics, clinical, and pathological features were well balanced between the NNAT and NAT cohorts, supporting comparability for subsequent outcome analyses.

**Table 1:** Patients baseline characteristics.

Characteristic	Overall (N = 80)	NNAT (N = 51)	NAT (N = 29)	*p*-Value
Age, years*, median (IQR)	67.5 (58.0, 72.5)	68.0 (61.0, 75.0)	66.0 (55.0, 70.0)	0.079
**BMI, mean ± SD**	22.3 ± 3.3	21.9 ± 3.0	23.0 ± 3.9	0.195
**Gender, n (%)**				>0.999
Male	69 (86.3%)	44 (86.3%)	25 (86.2%)	
Female	11 (13.8%)	7 (13.7%)	4 (13.8%)	
**Smoking, n (%)**				0.986
Yes	33 (41.3%)	21 (41.2%)	12 (41.4%)	
No	47 (58.8%)	30 (58.8%)	17 (58.6%)	
**Systemic disease**				
**Hypertension, n (%)**				0.814
Yes	29 (36.3%)	18 (35.3%)	11 (37.9%)	
No	51 (63.7%)	33 (64.7%)	18 (62.1%)	
**Diabetes, n (%)**				0.311
Yes	11 (13.8%)	9 (17.6%)	2 (6.9%)	
No	69 (86.3%)	42 (82.4%)	27 (93.1%)	
**Hydronephrosis, n (%)**				0.347
Yes	20 (25.0%)	11 (21.6%)	9 (31.0%)	
No	60 (75.0%)	40 (78.4%)	20 (69.0%)	
**Hepatitis, n (%)**				0.248
Yes	7 (8.8%)	3 (5.9%)	4 (13.8%)	
No	73 (91.3%)	48 (94.1%)	25 (86.2%)	
**Surgical methods, n (%)**				0.553
Orthotopic neobladder	8 (10.0%)	6 (11.8%)	2 (6.9%)	
Cutaneous stoma formation	32 (40.0%)	22 (43.1%)	10 (34.5%)	
Ileal conduit	40 (50.0%)	23 (45.1%)	17 (58.6%)	
**NAT protocol chemotherapy, n (%)**				<0.001
No	51 (63.7%)	51 (100.0%)	0 (0%)	
GC	27 (33.8%)	0 (0.0%)	27 (93.1%)	
GCa	2 (2.5%)	0 (0.0%)	2 (6.9%)	
**NAT protocol immunotherapy, n (%)**				<0.001
No	51 (63.7%)	51 (100.0%)	10 (34.5%)	
Tislelizumab	7 (8.8%)	0 (0.0%)	7 (24.1%)	
Toripalimab	12 (15.0%)	0 (0.0%)	12 (41.4%)	
**Clinical T stage, n (%)**				0.022
NMIBC (T1)	24 (30.0%)	20 (39.3%)	4 (13.8%)	
MIBC (T2–T4)	56 (70.0%)	31 (60.7%)	25 (86.2%)	
T2	19 (23.7%)	7 (13.7%)	12 (41.4%)	
T3	15 (18.8%)	9 (17.6%)	6 (20.7%)	
T4	22 (27.5%)	15 (29.4%)	7 (24.1%)	
**Clinical N stage, n (%)**				0.031
N0	70 (87.5%)	48 (94.1%)	22 (75.9%)	
N1	10 (12.5%)	3 (5.9%)	7 (24.1%)	
**Histological type, n (%)**				0.083
Pure urothelial carcinoma	65 (81.3%)	38 (74.5%)	27 (93.1%)	
Urothelial carcinoma with variant differentiation	11 (13.7%)	10 (19.6%)	1 (3.4%)	
Pure variant carcinoma	4 (5.0%)	3 (5.9%)	1 (3.4%)	
**Pathological grade, n (%)**				0.291
Low	4 (5.0%)	4 (7.8%)	0 (0%)	
High	76 (95.0%)	47 (92.2%)	29 (100.0%)	

**Abbreviations:** SD, standard deviation; IQR, inter-quartile range; NNAT, no neoadjuvant therapy; NAT, neoadjuvant therapy; BMI, body mass index; GC, gemcitabine + cisplatin; GCa, gemcitabine + carboplatin; **Notes:** *The rank-sum test (Mann-Whitney-U test) was used for this comparison because these data had non-normal distributions. Boldface text indicates the names of grouping variables. Histological type definitions: Pure urothelial carcinoma—invasive urothelial carcinoma without variant differentiation; Urothelial carcinoma with variant differentiation—includes squamous, glandular, or plasmacytoid differentiation. (One case of urothelial carcinoma with squamous differentiation also had concurrent carcinoma *in situ* (CIS); this case was not classified as mixed differentiation because CIS is not considered a variant differentiation.); Pure variant carcinoma—includes pure squamous cell carcinoma, sarcomatoid carcinoma, and small cell carcinoma.

### 3.2 Survival Outcomes of NAT Compared with NNAT after Radical Cystectomy

**Figure 1:**
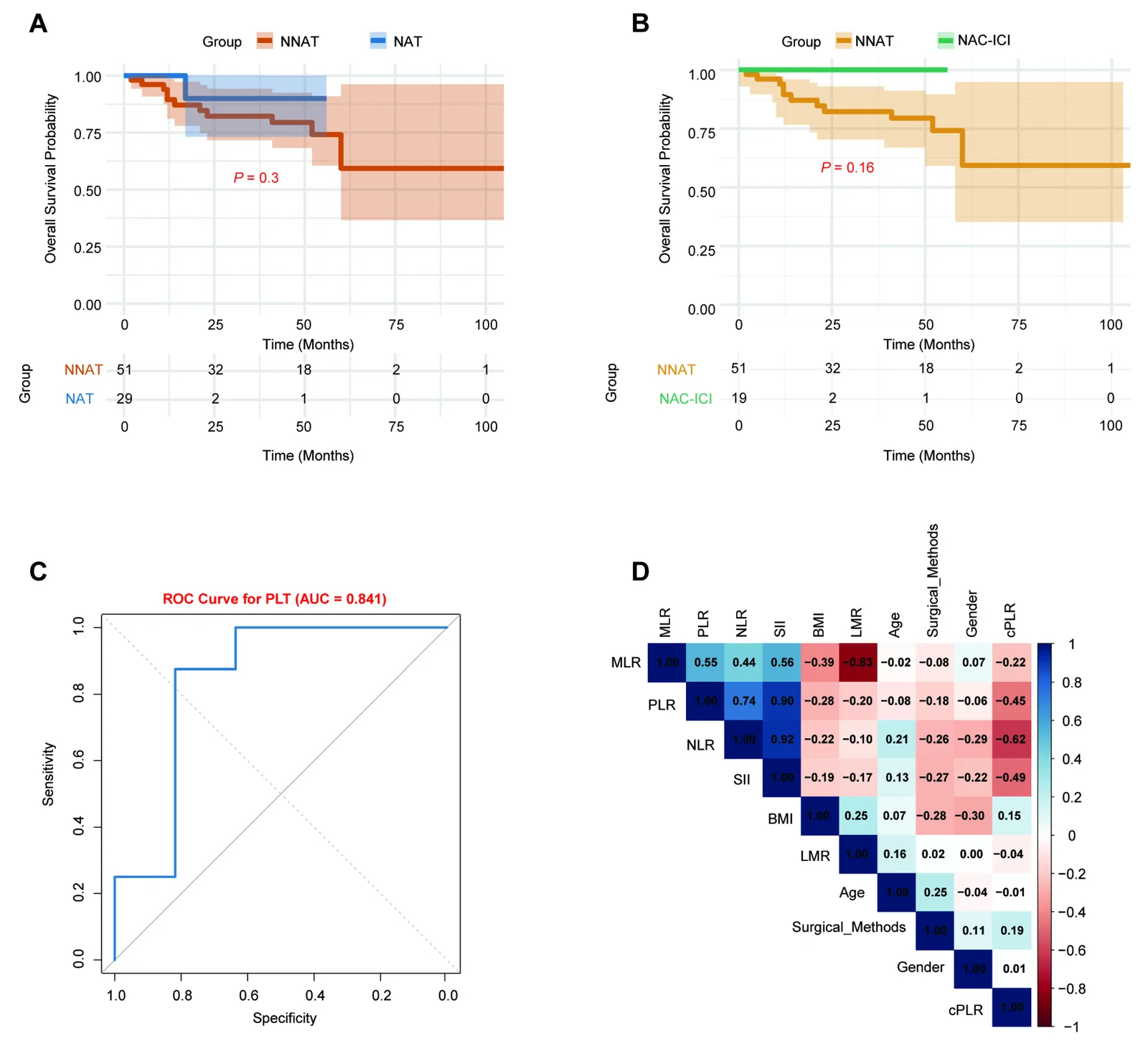
**Survival outcomes, predictive biomarker analysis, and correlations in patients undergoing radical cystectomy.** (**A**) Kaplan-Meier overall survival (OS) curves comparing patients who received neoadjuvant therapy (NAT, N = 29) with those who did not (no neoadjuvant therapy, NNAT; N = 51). NAT showed a numerically higher OS probability, although the difference did not reach statistical significance (log-rank *p* = 0.30); (**B**) Kaplan-Meier OS curves comparing NNAT (N = 51) with patients treated with immune checkpoint inhibitors with chemotherapy NAC-ICI (N = 19). A separation in survival curves was observed, favoring NAC-ICI, though the log-rank test did not show statistical significance (*p* = 0.16); (**C**) receiver operating characteristic (ROC) curve for platelet count (PLT) (area under the curve, AUC = 0.841). ROC curve for baseline PLT in predicting pathological complete response (pCR) to NAC-ICI. The AUC was 0.841, indicating good discriminatory ability; (**D**) Heatmap of correlations among hematological and clinical variables in the NAC-ICI cohort. Strong positive correlations were observed among neutrophil-to-lymphocyte ratio (NLR), platelet-to-lymphocyte (PLR), and systemic immune-inflammation index (SII).

Survival outcomes were compared to determine the prognostic benefit of NAT after radical cystectomy. Kaplan-Meier analysis illustrated that the survival curve of the NAT cohort was above that of the NNAT cohort (Fig. 1A), suggesting a potential survival advantage; however, the log-rank test did not demonstrate a statistically significant difference (χ^2^ = 1.10, *p* = 0.30). Median survival was not reached in either group. Given the limited number of events (NNAT: 11; NAT: 1), RMST was further evaluated up to the common follow-up limit (τ = 56 months). RMST was 47.73 months (95% CI, 42.89–52.57) in the NNAT cohort and 52.10 months (95% CI, 44.85–59.35) in the NAT cohort, corresponding to an absolute difference of +4.37 months (95% CI, −4.35 to +13.09; *p* = 0.33) and a ratio of 1.09 (95% CI, 0.92–1.30; *p* = 0.32). These findings indicated a non-significant trend favoring NAT, likely limited by the shorter follow-up period and the low event count in the NAT arm.

In subgroup analyses comparing NAC-ICI with NNAT (Fig. 1B), Kaplan-Meier curves were separated in favor of the NAC-ICI cohort. However, the log-rank test again did not reach statistical significance (χ^2^ = 2.0, *p* = 0.16), and median survival was not reached in either group. Given the absence of deaths in the NAC-ICI cohort (events: NNAT, 11; NAC-ICI, 0), RMST was additionally evaluated up to the common follow-up horizon (τ = 56 months). RMST was 47.73 months (95% CI, 42.89–52.57) in the NNAT cohort and 56.0 months (95% CI, 56–56) in the NAC-ICI cohort, yielding an absolute difference of +8.27 months (95% CI, 3.43–13.11; *p* = 0.0008) and a ratio of 1.17 (95% CI, 1.06–1.30; *p* = 0.002). Because no events occurred in the NAC-ICI arm, this absolute RMST difference is mathematically unstable and should be interpreted with caution. Meanwhile, Cox regression yielded unstable estimates due to zero events in the NAC-ICI cohort, further supporting the use of RMST as a more reliable measure of survival differences in this setting. Taken together, although survival curves suggested a potential benefit of NAT, particularly NAC-ICI, these findings did not reach statistical significance, most likely reflecting limited sample size and shorter follow-up rather than a true absence of effect.

### 3.3 Postoperative Complications Following Radical Cystectomy

Considering that the safety of NAT remains a critical concern in clinical decision-making, the influence of NAT on the incidence of postoperative complications after radical cystectomy was explored. As summarized in Table 2, no significant differences were observed between the NAT and NNAT cohorts across major complications, including paralytic ileus, deep vein thrombosis, anastomotic breakdown, urinary tract infection, and surgical site/wound complications (all *p* > 0.05). Specifically, paralytic ileus was observed in 13.7% of NNAT patients compared with 6.9% in NAT patients (*p* = 0.476). Deep vein thrombosis was noted in 11.8% versus 3.4% (*p* = 0.412) of patients, and anastomotic breakdown in 15.7% versus 3.4% (*p* = 0.145), respectively. Urinary tract infections were observed in 11.8% of NNAT patients and 13.8% of NAT patients (*p* > 0.999). At the same time, surgical site or wound complications were relatively rare, affecting 5.9% and 3.4% of patients, respectively (*p* > 0.999. These observations validated the clinical feasibility of NAT, indicating that its application does not impose additional surgical risks.

**Table 2:** Complications after radical cystectomy.

Complication	Overall (N = 80)	NNAT (N = 51)	NAT (N = 29)	*p*-Value
**Paralytic ileus**				0.476
Yes	9 (11.3%)	7 (13.7%)	2 (6.9%)	
No	71 (88.8%)	44 (86.3)	27 (93.1%)	
**Deep vein thrombosis**				0.412
Yes	7 (8.8%)	6 (11.8%)	1 (3.4%)	
No	73 (91.3%)	45 (88.2%)	28 (96.6%)	
**Anastomotic breakdown**				0.145
Yes	9 (11.3%)	8 (15.7%)	1 (3.4%)	
No	71 (88.8%)	43 (84.3%)	28 (96.6%)	
**Urinary tract infection**				>0.999
Yes	10 (12.5%)	6 (11.8%)	4 (13.8%)	
No	70 (87.5%)	45 (88.2%)	25 (86.2%)	
**Surgical site/wound complication**				>0.999
Yes	4 (5.0%)	3 (5.9%)	1 (3.4%)	
No	76 (95.0%)	48 (94.1%)	28 (96.6%)	

**Abbreviations:** NNAT: no neoadjuvant therapy; NAT: neoadjuvant therapy; **Note:** Boldface text indicates the names of grouping variables.

### 3.4 Pathologic Outcomes after Different Neoadjuvant Regimens

To evaluate the efficacy of different neoadjuvant approaches, pathologic responses were compared between patients treated with NAC and NAC-ICI (Table 3). Overall, 21 patients (72.4%) achieved pDS, including 10 (34.5%) who reached pCR. As anticipated, the pCR rate was higher in the NAC-ICI group than in the NAC group (42.1% vs. 20.0%), while pDS was similar (73.7% vs. 70.0%). However, neither comparison reached statistical significance (pCR, *p* = 0.414; pDS, *p* > 0.999). In the NAC-ICI cohort, eight patients achieved pCR and 11 did not, reflecting a higher absolute response rate compared to NAC alone. This finding indicates a favorable trend toward improved tumor clearance with chemoimmunotherapy, albeit without statistical significance.

**Table 3:** Pathologic response of two neoadjuvant schedules.

Response	Overall (N = 29)	NAC (N = 10)	NAC-ICI (N = 19)	*p*-Value
**pCR**	10 (34.5%)	2 (20.0%)	8 (42.1%)	0.414
**pDS**	21 (72.4%)	7 (70.0%)	14 (73.7%)	>0.999

**Abbreviations:** pCR, pathological complete response; pDS, pathological downstaging. NAC: neoadjuvant chemotherapy; NAC-ICI: immune checkpoint inhibitors with chemotherapy.

Furthermore, pathological responses were assessed by specific ICIs used within the NAC-ICI group (Table 4). The pCR rate was 58.3% for toripalimab and 14.3% for tislelizumab; the pDS rates were 83.3% and 57.1%, respectively. However, the sample size is very small (7 for tislelizumab, 12 for toripalimab). Therefore, no reliable comparison between the two agents can be made, and the data are presented only descriptively. Larger cohorts are needed to explore potential differences.

**Table 4:** Pathologic response of two immune checkpoint inhibitors.

Response	Overall (N = 19)	Tislelizumab (N = 7)	Toripalimab (N = 12)	*p*-Value
**pCR**	8 (42.1%)	1 (14.3%)	7 (58.3%)	0.147
**pDS**	14 (73.7%)	4 (57.1%)	10 (83.3%)	0.305

**Abbreviations:** pCR, pathological complete response; pDS, pathological downstaging.

### 3.5 Incidence and Spectrum of TRAEs

The safety profiles of NAC and NAC-ICI are listed in Table 5. Overall, the most common TRAEs of any grade were anemia and hepatic dysfunction. Anemia was identified in 80.0% of NAC patients and 94.7% of NAC-ICI patients, while hepatic dysfunction was observed in 40.0% and 42.1%, respectively. Other hematological toxicities, including lymphopenia (30.0% vs. 31.6%) and thrombocytopenia (0% vs. 10.5%), were observed at comparable rates. While neutropenia (5.3%), renal dysfunction (26.3%), and pruritus (5.3%) occurred only in the NAC-ICI cohort, they were infrequent. The rate of gastrointestinal toxicities such as nausea (30.0% vs. 26.3%) and vomiting (10.0% vs. 10.5%) was similar between groups. Notably, no grade IV events were reported, and most TRAEs were grade I-II. Overall, the addition of tislelizumab and toripalimab to chemotherapy did not significantly increase either the incidence or severity of TRAEs compared with chemotherapy alone.

**Table 5:** Treatment-related adverse events of two neoadjuvant schedules.

Adverse Event	NAC (N = 10)			NAC-ICI (N = 19)			*p*-Value
	Grade I–II	Grade III–IV	Incidence (%)	Grade I–II	Grade III–IV	Incidence (%)	
**Blood and lymphatic system disorders**							
Anemia	7	1	80.0	16	2	94.7	0.267
Neutropenia	-	-	-	1	-	5.3	>0.999
Thrombocytopenia	-	-	-	2	-	10.5	0.532
Lymphopenia	3	-	30.0	5	1	31.6	>0.999
Leukopenia	-	-	-	-	-	-	-
**Hepatic dysfunction**	4	-	40.0	8	-	42.1	>0.999
**Renal dysfunction**	-	-	-	5	-	26.3	0.134
**Gastrointestinal disorders**							
Nausea	3	-	30.0	5	-	26.3	>0.999
Vomiting	1	-	10.0	2	-	10.5	>0.999
**Skin and subcutaneous tissue disorders**							
Pruritus	-	-	-	1	-	5.3	>0.999

**Abbreviations:** NAC: neoadjuvant chemotherapy; NAC-ICI: immune checkpoint inhibitors with chemotherapy. **Note:** Boldface text indicates the names of grouping variables.

### 3.6 Exploratory Biomarker Analysis for pCR in NAC-ICI Cohort

The NAC-ICI cohort exhibited a numerically higher pCR rate, suggesting potential therapeutic benefit. Nevertheless, reliable biomarkers for predicting response to NAC-ICI remain to be identified. Univariate analysis of baseline clinicopathological variables (Table 6) revealed a significant difference in platelet count (PLT) between pCR responders and non-responders (*p* = 0.005). More importantly, logistic regression validated that elevated baseline PLT was significantly associated with a reduced likelihood of achieving pCR (OR = 0.977, 95% CI: 0.949–0.994, *p* = 0.042). ROC analysis further supported the predictive role of PLT, with an area under the curve (AUC) of 0.841 (95% CI: 0.653–1.000, *p* = 0.013) (Fig. 1C). The optimal cut-off value for PLT was 259 × 10^9^/L, at which sensitivity reached 81.8% and specificity 87.5%, indicating robust discriminative performance. These findings suggest that baseline PLT may serve as a practical biomarker to stratify patients likely to achieve pCR following NAC-ICI.

To elucidate the interplay among inflammatory markers, correlation analysis was conducted across 11 variables (Fig. 1D). Of note, strong positive correlations were observed among NLR, PLR, and SII (NLR-PLR, R = 0.74; NLR-SII, R = 0.92; PLR-SII, R = 0.90), highlighting their synergistic role in reflecting systemic inflammatory and immune activation states. This suggests that neutrophil and platelet proliferation, along with relative lymphocyte depletion, collectively drive changes in these ratios. In the clinical setting, either a single marker or a composite of these indices may provide equivalent insight into the inflammatory status. In contrast, demographic features (age, gender) and surgical methods showed minimal correlations with inflammatory indices (|R| < 0.3), indicating that hematologic fluctuations were primarily immune-related rather than patient- or procedure-driven in this cohort.

**Table 6:** Univariate analysis of pathological response-related factors of NAC-ICI cohort.

Variable	Overall (N = 19)	pCR (N = 8)	Non-pCR (N = 11)	*p*-Value
**Clinical stage**				>0.999
T2N0M0	10 (52.6%)	4 (50.0%)	6 (54.6%)	
>T2N0M0	7 (36.8%)	3 (37.5%)	4 (36.4%)	
**Histological type**				0.421
Pure urothelial carcinoma	18 (94.7%)	7 (87.5%)	11 (100.0%)	
Urothelial carcinoma with variant differentiationr	0 (0.0%)	0 (0.0%)	0 (0.0%)	
Pure variant or mixed carcinoma	1 (5.3%)	1 (12.5%)	0 (0.0%)	
**Hematological parameters**				
**Erythrocyte, mean ± SD**	4.07 ± 0.64	4.07 ± 0.40	4.07 ± 0.80	0.983
**Leukocyte, mean ± SD**	8.98 ± 3.78	8.46 ± 3.88	9.35 ± 3.85	0.628
**Neutrophil, mean ± SD**	6.90 ± 3.60	6.58 ± 3.67	7.14 ± 3.71	0.748
**Lymphocyte, mean ± SD**	1.35 ± 0.54	1.27 ± 0.61	1.40 ± 0.51	0.630
Eosinophil*, median (IQR)	0.13 (0.04, 0.23)	0.13 (0.01, 0.25)	0.13 (0.85, 0.19)	0.840
Monocyte*, median (IQR)	0.37 (0.31, 0.71)	0.31 (0.23, 0.40)	0.57 (0.35, 0.81)	0.075
**Platelet, mean ± SD**	269.89 ± 93.12	207.88 ± 47.80	315.00 ± 93.24	0.005
**Hemoglobin, mean ± SD**	113.00 ± 22.34	120.75 ± 10.40	107.36 ± 27.19	0.159
**Albumin, mean ± SD**	36.08 ± 3.80	35.60 ± 3.82	36.44 ± 3.92	0.648
NLR*, median (IQR)	4.34 (3.28, 9.11)	4.34 (2.78, 10.56)	4.34 (3.33, 7.42)	>0.999
PLR*, median (IQR)	202.02 (140.60, 299.41)	168.49 (110.30, 299.41)	204.90 (164.54, 285.49)	0.272
SII*, median (IQR)	1058.33 (721.35, 2463.79)	1038.29 (477.68, 2456.19)	1270.35 (848.26, 2463.79)	0.492
MLR*, median (IQR)	0.35 (0.25, 0.47)	0.29 (0.17, 0.45)	0.40 (0.28, 0.57)	0.142
LMR*, median (IQR)	2.89 (2.17, 4.05)	3.51 (2.24, 6.05)	2.51 (1.86, 3.56)	0.153
cPLR*, mean ± SD	0.43 ± 0.59	0.26 ± 0.49	0.55 ± 0.65	0.717

**Abbreviations:** SD, standard deviation; IQR, inter-quartile range; pCR, pathological complete response; non-pCR, no pathological complete response; NLR, neutrophil-to-lymphocyte ratio; PLR, platelet-to-lymphocyte ratio; SII, systemic immune-inflammation index, equals to [platelet count × neutrophil count]/lymphocyte count; MLR, monocyte-to-lymphocyte ratio; LMR, lymphocyte-to-monocyte ratio; cPLR, corrected platelet-to-lymphocyte ratio, equals to [post-therapy or intra-therapy PLR-pre-therapy PLR]/pre-therapy PLR. **Notes:** *The rank-sum test (Mann-Whitney-U test) was used for this comparison because these data had non-normal distributions. Boldface text indicates the names of grouping variables.

## 4 Discussion

Although radical cystectomy remains the gold-standard treatment for MIBC, the high risk of micrometastatic disease highlights the need for effective systemic therapy [31]. This study evaluated the clinical utility of NAT in a retrospective cohort of patients undergoing radical cystectomy. Kaplan-Meier curves displayed a numerically higher survival probabilities in the NAT cohort compared with NNAT. However, the log-rank test did not reach statistical significance (*p* = 0.30), likely ascribed to the small sample size (N = 29 in the NAT group) and limited follow-up. To address the low event rate, RMST analysis was applied, which demonstrated a non-significant trend for NAT (52.10 vs. 47.73 months; Δ = +4.37 months; *p* = 0.33). These findings do not rule out a survival benefit, though the current study lacked sufficient power for drawing definitive conclusions. 

Pathological evaluations uncovered that the NAT cohort achieved a pCR rate of 34.5% and a pDS rate of 72.4%. Noteworthily, the pCR rate was lower compared to earlier studies [32,33,34,35], whereas the pDS rate was comparable or higher [32,33]. The relatively modest pCR rate may be attributed to the higher proportion of patients with advanced-stage disease (≥T3N0M0, 44.8%), which has been consistently reported as an unfavorable factor for achieving pathologic remission [36,37]. These findings underscore the heterogeneity of response in real-world cohorts compared with trial populations.

Beyond clinical and pathologic outcomes, the identification of biomarkers that can predict treatment response and guide personalized therapy has garnered extensive attention. Among various candidate biomarkers, including genetic, immunological, and hematological factors, systemic inflammatory markers derived from peripheral blood have attracted attention owing to their accessibility and prognostic value across multiple malignancies [38]. For example, elevated pre-treatment NLR, PLR, SII, and MLR, or reduced LMR, have been associated with poor survival outcomes in patients with bladder cancer undergoing radical cystectomy [39,40,41,42,43]. Similarly, a high PLT has been identified as a negative prognostic factor in gastrointestinal and gynecologic cancers [44,45,46,47].

Herein, baseline PLT emerged as the only significant hematological predictor of pathologic response to NAC-ICI. While other inflammatory ratios (NLR, PLR, SII, MLR, and LMR) showed no significant associations with pCR, higher baseline PLT was negatively correlated with tumor eradication. It is worthwhile emphasizing that logistic regression corroborated this association (OR = 0.977, 95% CI: 0.949–0.994, *p* = 0.042), and ROC analysis demonstrated favorable predictive performance (AUC = 0.841, 95% CI: 0.653–1.000, *p* = 0.013). A cutoff value of 259 × 10^9^/L maximized the Youden index (J = 0.693), achieving 81.8% sensitivity and 87.5% specificity for predicting pCR. These findings imply that elevated baseline PLT may impair pathological response to NAC-ICI, although validation in larger, prospective cohorts is required to establish its predictive utility.

Circulating tumor DNA (ctDNA) has emerged as a promising non-invasive biomarker in muscle-invasive bladder cancer. Detection of tumor DNA in plasma and urine before neoadjuvant chemotherapy is associated with a lower response rate, and post-treatment detection correlates with worse oncological outcomes [48]. Moreover, ctDNA status before radical cystectomy predicts recurrence-free survival, and ctDNA clearance during neoadjuvant therapy is predictive of treatment response [49]. Although our study did not include ctDNA assessment, these findings underscore its potential to guide patient selection and perioperative treatment decisions. Future prospective trials integrating ctDNA dynamics with conventional clinicopathological and hematological markers are warranted.

The safety profile of NAT is a key consideration for its clinical application. The safety analysis of the two neoadjuvant regimens in this study demonstrated that both the NAC and NAC-ICI regimens exhibited manageable safety profiles, with adverse reactions largely of grade I–II events, and grade III–IV events being rare. This overall tolerability profile is more favorable compared to data from previous studies investigating platinum-based chemotherapy regimens [50]. Hematological toxicity was the most common TRAE in both groups. The incidence of anemia of all grades was 94.7% in the NAC-ICI group and 80.0% in the NAC group. Although anemia is an established toxicity of platinum-based chemotherapy [51], its incidence was higher in the NAC-ICI group, indicating that, in addition to the myelosuppressive effect of chemotherapy, PD-1 inhibitors tislelizumab and toripalimab may exert a marginal superimposed effect on erythropoiesis [52], although this difference was not statistically significant herein. Renal dysfunction was exclusively observed in the NAC-ICI group, with an incidence rate as high as 26.3% (all grades I–II), whereas no events were observed in the NAC group, but the difference was not statistically significant (*p* = 0.134). The occurrence of this phenomenon may be influenced by multiple factors. Firstly, cisplatin has definite dose-dependent nephrotoxicity, a primary dose-limiting toxicity [53,54]. Compared with cisplatin, the non-hematological toxicity of carboplatin, especially nephrotoxicity, is significantly lower [55]. The two groups of NAT patients in the present study were highly comparable in chemotherapy regimens. Specifically, nine patients (90%) received cisplatin, and one patient (10%) was administered carboplatin in the NAC group, whereas 18 cases (94.7%) received cisplatin and one case (5.3%) received carboplatin in the NAC-ICI group. Thus, the underlying risk for cisplatin-related nephrotoxicity was nearly identical between the two groups. However, no nephrotoxic events were observed in the NAC group, while the incidence rate was 26.3% in the NAC-ICI group.

This pronounced difference suggests that the use of carboplatin in one case in the NAC group may have played a decisive role in diluting the overall nephrotoxicity profile. Secondly, PD-1 inhibitors such as tislelizumab and toripalimab can elicit immune-related adverse events such as acute kidney injury [56]. Therefore, their combination with platinum-based nephrotoxic drugs may exacerbate renal injury [57]. Finally, the NAC-ICI group included two PD-1 inhibitors (toripalimab, N = 12; tislelizumab, N = 7). Although their adverse event profiles are hypothesized to be generally similar, subtle differences in drug-specific toxicity remain confounding factors that cannot be completely ruled out [58]. The safety data of this study indicated that despite highly similar background chemotherapy regimens (composed of cisplatin/carboplatin), renal toxicity signals were observed after combination with PD-1 inhibitors. This finding strongly suggests that the addition of PD-1 inhibitors is the primary factor leading to the increased incidence of renal dysfunction, potentially through mechanisms independent or synergistic with platinum-induced nephrotoxicity.

Nevertheless, several limitations of this study should be acknowledged. To begin, its retrospective and single-center design might have introduced selection bias and limited the generalizability of the findings. Secondly, the relatively small sample size, particularly in the NAT subgroup, coupled with the low number of survival events, substantially reduced the statistical power of survival analyses and increased the risk of type II error. Thirdly, the follow-up period was limited, which precluded robust estimation of long-term survival outcomes and may have underestimated late adverse events. Fourthly, heterogeneity in NAT regimens, including the use of two different PD-1 inhibitors (tislelizumab and toripalimab) and variations in platinum agents (cisplatin vs. carboplatin), complicates the interpretation of efficacy and safety signals. Fifthly, the distribution of clinical N stage (N0 vs. N1) was not balanced between the NNAT and NAT groups (*p* = 0.031), with more node-positive patients in the NAT group. This imbalance may have confounded the survival comparison, and we did not adjust for N stage due to the small number of node-positive cases. Sixthly, the exploratory biomarker analysis was constrained by the relatively limited sample size and absence of external validation, necessitating cautious interpretation. Finally, post-operative maintenance immunotherapy, if any, was not consistently recorded in this retrospective study; therefore, its potential impact on overall survival could not be assessed.

Of note, this real-world study provides several complementary insights: (i) a head-to-head comparison of NAC, NAC-ICI, and upfront radical cystectomy; (ii) inclusion of high-risk T1 patients; (iii) use of RMST to handle low event rates; and (iv) an exploratory analysis of PLT as a predictive biomarker. These features underscore the feasibility and safety of neoadjuvant chemoimmunotherapy in routine clinical practice.

## 5 Conclusions

Despite the limitations inherent to a single-center retrospective design, this study provides valuable real-world evidence supporting the feasibility, safety, and potential clinical benefit of NAC in bladder cancer, particularly when combining chemotherapy with PD-1 inhibitors. The observed trends in survival improvement and pathological response, along with the identification of PLT as a potential exploratory biomarker, highlight promising directions for future research. Larger, prospective, multi-center studies with extended follow-up are warranted to validate these findings and to refine patient selection strategies for NAC-ICI in bladder cancer.

## Data Availability

The raw data supporting the conclusions of this article will be made available from the corresponding author upon reasonable request.
